# Supplementary material for: Stable body size of Alpine ungulates
Source: R Soc Open Sci. 2020 Jul 15;7(7):200196. doi: 10.1098/rsos.200196 (PMC7428221; doi:10.1098/rsos.200196)
Supplement: Supplementary Material (Buentgen_etal.2020_sm) [file rsos200196supp1.docx]

**Supplementary Materials (Table S1–S4, Figs. S1–S10)**

**Stable body size of Alpine ungulates**

Ulf Büntgen,^1,2*^ Hannes Jenny,^3^ J. Diego Galván,^2^ Alma Piermattei,^1^ Paul J. Krusic,^1,4^ and Kurt Bollmann^2^

*^1^Department of Geography, University of Cambridge, Downing Place, CB2 3EN, UK*

*^2^Swiss Federal Research Institute WSL, Zürcherstr 111, 8903 Birmensdorf, Switzerland*

*^3^Department of Wildlife and Fishery Service Grisons, Loëstrasse 14, 7001 Chur, Switzerland*

*^4^Department of Physical Geography, Stockholm University, SE-10691, Stockholm, Sweden*

*^*^Author for Correspondence: ulf.buentgen@geog.cam.ac.uk (Ulf Büntgen)*

**Table S1.** Stepwise multiple regression analysis of traits and other variables of male and female ibex (*Capra ibex*), which were harvested in Grisons between 1991 and 2013. Eviscerated body weight (EBW), hint foot length (HFL) and lower jaw length (LJL) were used as dependent variables, whereas animal age, harvest elevation, harvest year, monthly mean temperature and monthly NDVI were used as independent variables. After adjusting R^2^ for different sample sizes, all models reveal significant estimates (**P <0.01, ***P <0.001).

|  | | **FEMALE** | | | | **MALE** | | | |
| --- | --- | --- | --- | --- | --- | --- | --- | --- | --- |
|  |  | **Parameters** | **Estimate** | **Std. Error** | **t value** | **Parameters** | **Estimate** | **Std. Error** | **t value** |
| **IBEX** | **EBW** | **Adj R^2^ = 0.552*****  **No. obs. = 7273** |  |  |  | **Adj R^2^ =0.825*****  **No. obs. = 5970** |  |  |  |
|  |  | Intercept *** | 1.09E+00 | 6.04E-01 | 1.813 | Intercept *** | 3.73E+00 | 1.92E-02 | 194.58 |
|  |  | Animal Age *** | 2.49E-01 | 2.77E-03 | 89.762 | Animal Age *** | 4.74E-01 | 2.96E-03 | 160.27 |
|  |  | I(Animal Age^2) *** | -1.20E-01 | 2.02E-03 | -59.4 | I(Animal Age^2) *** | -1.52E-01 | 1.85E-03 | -81.89 |
|  |  | Hunting Elevation *** | 9.26E-05 | 5.84E-06 | 15.867 | Hunting Elevation *** | 7.81E-05 | 7.45E-06 | 10.48 |
|  |  | T_May *** | 7.91E-03 | 1.87E-03 | 4.238 |  |  |  |  |
|  |  | Hunting Year *** | 1.03E-03 | 3.03E-04 | 3.41 |  |  |  |  |
|  | **HFL** | **Adj R^2^ = 0.211*****  **No. obs. = 7248** |  |  |  | **Adj R^2^ =0.474*****  **No. obs. = 5962** |  |  |  |
|  |  | Intercept *** | 2.86E+00 | 1.54E-01 | 18.652 | Intercept *** | 3.49E+00 | 5.17E-03 | 674.824 |
|  |  | Animal Age *** | 3.10E-02 | 7.16E-04 | 43.293 | Animal Age *** | 5.64E-02 | 7.92E-04 | 71.182 |
|  |  | I(Animal Age^2) *** | -1.34E-02 | 5.23E-04 | -25.677 | I(Animal Age^2) *** | -2.19E-02 | 4.98E-04 | -43.963 |
|  |  | Hunting Year *** | 2.60E-04 | 7.67E-05 | 3.388 | Hunting Elevation *** | 9.10E-06 | 2.01E-06 | 4.539 |
|  | **LJL** | **Adj R^2^ = 0.578*****  **No. obs. = 3216** |  |  |  | **Adj R^2^ =0.603*****  **No. obs. = 2253** |  |  |  |
|  |  | Intercept *** | 0.7696745 | 0.631308 | 1.219 | Intercept *** | -0.3597843 | 0.6691735 | -0.538 |
|  |  | Animal Age *** | 0.0924326 | 0.0014237 | 64.923 | Animal Age *** | 0.0877928 | 0.0015153 | 57.936 |
|  |  | I(Animal Age^2) *** | -0.0364618 | 0.0010374 | -35.147 | I(Animal Age^2) *** | -0.034385 | 0.0011027 | -31.184 |
|  |  | Hunting Year *** | 0.001063 | 0.0003158 | 3.366 | Hunting Year *** | 0.0016628 | 0.0003347 | 4.968 |

**Table S2.** Stepwise multiple regression analysis of traits and other variables of male and female chamois (*Rupicapra rupicapra*), which were harvested in Grisons between 1991 and 2013. Eviscerated body weight (EBW), hint foot length (HFL) and lower jaw length (LJL) were used as dependent variables, whereas animal age, harvest elevation, harvest year, monthly mean temperature and monthly NDVI were used as independent variables. After adjusting R2 for different sample sizes, all models reveal significant estimates (**P <0.01, ***P <0.001).

|  | | **FEMALE** | | | | **MALE** | | | |
| --- | --- | --- | --- | --- | --- | --- | --- | --- | --- |
|  |  | **Parameters** | **Estimate** | **Std. Error** | **t value** | **Parameters** | **Estimate** | **Std. Error** | **t value** |
| **CHAMOIS** | **EBW** | **Adj R^2^ = 0.452*****  **No. obs. = 46090** |  |  |  | **Adj R^2^ =0.669*****  **No. obs. = 37999** |  |  |  |
|  |  | Intercept *** | 2.99E+00 | 3.07E-03 | 972.185 | Intercept *** | 4.98E+00 | 2.62E-01 | 19.032 |
|  |  | Animal Age *** | 1.85E-01 | 9.99E-04 | 184.726 | Animal Age *** | 2.61E-01 | 9.98E-04 | 261.541 |
|  |  | I(Animal Age^2) *** | -9.96E-02 | 7.46E-04 | -133.618 | I(Animal Age^2) *** | -1.15E-01 | 6.42E-04 | -178.733 |
|  |  | Hunting Elevation *** | 2.65E-05 | 1.44E-06 | 18.422 | Hunting Elevation *** | 2.69E-05 | 1.80E-06 | 14.985 |
|  |  | T_Mar *** | 5.52E-03 | 6.99E-04 | 7.897 | Hunting Year *** | -8.58E-04 | 1.31E-04 | -6.557 |
|  |  |  |  |  |  | T_Mar *** | 4.17E-03 | 8.35E-04 | 4.989 |
|  | **HFL** | **Adj R^2^ = 0.158*****  **No. obs. = 32114** |  |  |  | **Adj R^2^ =0.278*****  **No. obs. = 25610** |  |  |  |
|  |  | Intercept *** | 2.81E+00 | 6.75E-02 | 41.574 | Intercept *** | 3.60E+00 | 1.17E-03 | 3087.05 |
|  |  | Animal Age *** | 2.40E-02 | 3.15E-04 | 76.278 | Animal Age *** | 3.06E-02 | 3.12E-04 | 98.38 |
|  |  | I(Animal Age^2) *** | -1.09E-02 | 2.29E-04 | -47.344 | I(Animal Age^2) *** | -1.30E-02 | 2.02E-04 | -64.65 |
|  |  | Hunting Elevation *** | -1.10E-05 | 4.60E-07 | -23.93 | Hunting Elevation *** | -9.02E-06 | 5.65E-07 | -15.97 |
|  |  | Hunting Year *** | 3.73E-04 | 3.37E-05 | 11.071 | T_Mar *** | 1.18E-03 | 2.66E-04 | 4.44 |
|  |  | T_Mar *** | 1.06E-03 | 2.28E-04 | 4.646 |  |  |  |  |
|  | **LJL** | **Adj R^2^ = 0.492***;**  **No. obs. = 22311** |  |  |  | **Adj R^2^ =0.469*****  **No. obs. = 18301** |  |  |  |
|  |  | Intercept *** | 2.7283525 | 0.0004933 | 5530.624 | Intercept *** | 4.46E+00 | 1.41E-01 | 31.689 |
|  |  | Animal Age *** | 0.0634701 | 0.0004475 | 141.836 | Animal Age *** | 5.16E-02 | 4.36E-04 | 118.23 |
|  |  | I(Animal Age^2) *** | -0.0257986 | 0.0003519 | -73.322 | I(Animal Age^2) *** | -2.06E-02 | 2.86E-04 | -72.097 |
|  |  | T_Jun *** | -0.0023243 | 0.0003901 | -5.958 | Hunting Year *** | -8.62E-04 | 7.04E-05 | -12.251 |
|  |  | NDVI_May *** | 0.0164625 | 0.0012521 | 13.148 | Hunting Elevation *** | 7.30E-06 | 7.79E-07 | 9.36 |
|  |  | NDVI_Jun *** | -0.005281 | 0.000729 | -7.244 | NDVI_May *** | 1.23E-02 | 1.24E-03 | 9.927 |
|  |  |  |  |  |  | NDVI_Apr *** | -1.06E-02 | 1.27E-03 | -8.287 |
|  |  |  |  |  |  | T_May *** | -2.12E-03 | 4.88E-04 | -4.339 |

**Table S3.** Stepwise multiple regression analysis of traits and other variables of male and female red deer (*Cervus elaphus*), which were harvested in Grisons between 1991 and 2013. Eviscerated body weight (EBW), hint foot length (HFL) and lower jaw length (LJL) were used as dependent variables, whereas animal age, harvest elevation, harvest year, monthly mean temperature and monthly NDVI were used as independent variables. After adjusting R2 for different sample sizes, all models reveal significant estimates (**P <0.01, ***P <0.001).

|  | | **FEMALE** | | | | **MALE** | | | |
| --- | --- | --- | --- | --- | --- | --- | --- | --- | --- |
|  |  | **Parameters** | **Estimate** | **Std. Error** | **t value** | **Parameters** | **Estimate** | **Std. Error** | **t value** |
| **RED DEER** | **EBW** | **Adj R^2^ = 0.444*****  **No. obs. = 28837** |  |  |  | **Adj R^2^ =0.644*****  **No. obs. = 36214** |  |  |  |
|  |  | Intercept *** | 6.19E+00 | 2.17E-01 | 28.554 | Intercept *** | 7.01E+00 | 2.82E-01 | 24.879 |
|  |  | Animal Age *** | 1.78E-01 | 1.36E-03 | 130.589 | Animal Age *** | 2.95E-01 | 1.26E-03 | 233.365 |
|  |  | I(Animal Age^2) *** | -4.52E-02 | 5.97E-04 | -75.758 | I(Animal Age^2) *** | -5.18E-02 | 4.34E-04 | -119.262 |
|  |  | Hunting Elevation *** | -3.90E-05 | 1.85E-06 | -21.107 | Hunting Elevation *** | -5.92E-05 | 2.02E-06 | -29.264 |
|  |  | Hunting Year *** | -9.84E-04 | 1.08E-04 | -9.079 | Hunting Year *** | -1.19E-03 | 1.41E-04 | -8.434 |
|  |  | T_Mar *** | 4.94E-03 | 7.44E-04 | 6.637 | T_May *** | -3.47E-03 | 9.00E-04 | -3.861 |
|  |  |  |  |  |  | NDVI_Mar *** | 3.61E-03 | 8.59E-04 | 4.203 |
|  |  |  |  |  |  | T_Apr *** | -3.37E-03 | 9.72E-04 | -3.469 |
|  | **HFL** | **Adj R^2^ = 0.129*****  **No. obs. = 27545** |  |  |  | **Adj R^2^ =0.165*****  **No. obs. = 34866** |  |  |  |
|  |  | Intercept *** | 3.91E+00 | 1.00E-03 | 3894.36 | Intercept *** | 3.96E+00 | 9.75E-04 | 4061.006 |
|  |  | Animal Age *** | 2.33E-02 | 4.55E-04 | 51.25 | Animal Age *** | 2.78E-02 | 3.57E-04 | 77.934 |
|  |  | I(Animal Age^2) *** | -5.79E-03 | 1.99E-04 | -29.14 | I(Animal Age^2) *** | -5.75E-03 | 1.23E-04 | -46.713 |
|  |  | Hunting Elevation *** | -1.29E-05 | 6.19E-07 | -20.82 | Hunting Elevation *** | -9.40E-06 | 5.69E-07 | -16.519 |
|  |  |  |  |  |  | NDVI_Jun *** | -1.01E-03 | 2.32E-04 | -4.331 |
|  | **LJL** | **Adj R^2^ = 0.593*****  **No. obs. = 28352** |  |  |  | **Adj R^2^ =0.493*****  **No. obs. = 35183** |  |  |  |
|  |  | Intercept *** | 1.70E+00 | 9.24E-02 | 18.418 | Intercept *** | 1.77E+00 | 8.53E-02 | 20.801 |
|  |  | Animal Age *** | 9.86E-02 | 5.51E-04 | 179.039 | Animal Age *** | 7.69E-02 | 4.44E-04 | 173.253 |
|  |  | I(Animal Age^2) *** | -2.53E-02 | 2.41E-04 | -104.827 | I(Animal Age^2) *** | -1.48E-02 | 1.53E-04 | -96.831 |
|  |  | Hunting Year *** | 7.99E-04 | 4.62E-05 | 17.295 | Hunting Year *** | 7.98E-04 | 4.26E-05 | 18.708 |
|  |  | Hunting Elevation *** | -9.96E-06 | 7.45E-07 | -13.362 | Hunting Elevation *** | -9.27E-06 | 7.12E-07 | -13.032 |
|  |  | T_Jun *** | 1.61E-03 | 3.19E-04 | 5.041 | NDVI_Mar *** | 1.46E-03 | 2.89E-04 | 5.049 |

**Table S4.** Stepwise multiple regression analysis of traits and other variables of male and female roe deer (*Capreolus capreolus*), which were harvested in Grisons between 1991 and 2013. Eviscerated body weight (EBW), hint foot length (HFL) and lower jaw length (LJL) were used as dependent variables, whereas animal age, harvest elevation, harvest year, monthly mean temperature and monthly NDVI were used as independent variables. After adjusting R2 for different sample sizes, all models reveal significant estimates (**P <0.01, ***P <0.001).

|  | | **FEMALE** | | | | **MALE** | | | |
| --- | --- | --- | --- | --- | --- | --- | --- | --- | --- |
|  |  | **Parameters** | **Estimate** | **Std. Error** | **t value** | **Parameters** | **Estimate** | **Std. Error** | **t value** |
| **ROE DEER** | **EBW** | **Adj R^2^ = 0.146*****  **No. obs. = 23986** |  |  |  | **Adj R^2^ =0.199*****  **No. obs. = 32875** |  |  |  |
|  |  | Intercept *** | 2.76E+00 | 2.70E-03 | 1024.649 | Intercept *** | 2.86E+00 | 2.32E-03 | 1231.886 |
|  |  | Animal Age *** | 6.70E-02 | 1.16E-03 | 57.817 | Animal Age *** | 6.65E-02 | 7.47E-04 | 89.005 |
|  |  | I(Animal Age^2) *** | -2.16E-02 | 5.93E-04 | -36.398 | I(Animal Age^2) *** | -2.03E-02 | 3.53E-04 | -57.482 |
|  |  | Hunting Elevation *** | 3.20E-05 | 1.70E-06 | 18.878 | Hunting Elevation *** | 9.96E-06 | 1.43E-06 | 6.988 |
|  |  | T_Apr *** | -5.91E-03 | 7.93E-04 | -7.46 | NDVI_Mar *** | 3.77E-03 | 7.23E-04 | 5.212 |
|  |  | NDVI_Mar *** | 5.72E-03 | 9.52E-04 | 6.012 | NDVI_Jun *** | -3.02E-03 | 6.72E-04 | -4.502 |
|  |  | NDVI_Jun *** | -2.79E-03 | 8.68E-04 | -3.21 |  |  |  |  |
|  | **HFL** | **Adj R^2^ = 0.037*****  **No. obs. = 13919** |  |  |  | **Adj R^2^ =0.019*****  **No. obs. = 19879** |  |  |  |
|  |  | Intercept *** | 2.99E+00 | 9.43E-02 | 31.708 | Intercept *** | 3.56E+00 | 1.03E-03 | 3456.748 |
|  |  | Animal Age *** | 9.42E-03 | 4.52E-04 | 20.85 | Animal Age *** | 5.88E-03 | 3.19E-04 | 18.437 |
|  |  | I(Animal Age^2) *** | -2.78E-03 | 2.32E-04 | -11.941 | I(Animal Age^2) *** | -1.70E-03 | 1.49E-04 | -11.412 |
|  |  | Hunting Elevation *** | -3.10E-06 | 6.55E-07 | -4.723 | Hunting Elevation *** | -4.16E-06 | 6.19E-07 | -6.716 |
|  |  | T_Mar *** | 1.01E-03 | 2.97E-04 | 3.397 | NDVI_Jun *** | -1.23E-03 | 2.57E-04 | -4.782 |
|  |  | Hunting Year *** | 2.81E-04 | 4.71E-05 | 5.976 |  |  |  |  |
|  |  | T_Apr *** | -1.48E-03 | 3.26E-04 | -4.534 |  |  |  |  |
|  | **LJL** | **Adj R^2^ = 0.189*****  **No. obs. = 14595** |  |  |  | **Adj R^2^ =0.144*****  **No. obs. = 18898** |  |  |  |
|  |  | Intercept *** | 2.73E+00 | 1.15E-03 | 2363.463 | Intercept *** | 3.28E+00 | 1.02E-01 | 32.272 |
|  |  | Animal Age *** | 2.52E-02 | 4.91E-04 | 51.416 | Animal Age *** | 1.98E-02 | 3.71E-04 | 53.402 |
|  |  | I(Animal Age^2) *** | -6.62E-03 | 2.45E-04 | -27.042 | I(Animal Age^2) *** | -5.46E-03 | 1.81E-04 | -30.167 |
|  |  | Hunting Elevation *** | 5.33E-06 | 7.33E-07 | 7.269 | NDVI_Jun *** | 3.88E-03 | 3.46E-04 | 11.231 |
|  |  | T_Apr *** | -3.73E-03 | 5.17E-04 | -7.213 | T_Jun *** | -2.32E-03 | 3.47E-04 | -6.677 |
|  |  | T_Mar *** | -1.74E-03 | 3.64E-04 | -4.779 | Hunting Year *** | -2.69E-04 | 5.08E-05 | -5.3 |
|  |  | NDVI_May *** | 1.39E-02 | 1.08E-03 | 12.804 | Hunting Elevation *** | 2.58E-06 | 6.88E-07 | 3.744 |
|  |  | NDVI_Apr *** | -1.05E-02 | 1.13E-03 | -9.293 |  |  |  |  |
|  |  | T_May *** | -1.70E-03 | 3.97E-04 | -4.274 |  |  |  |  |


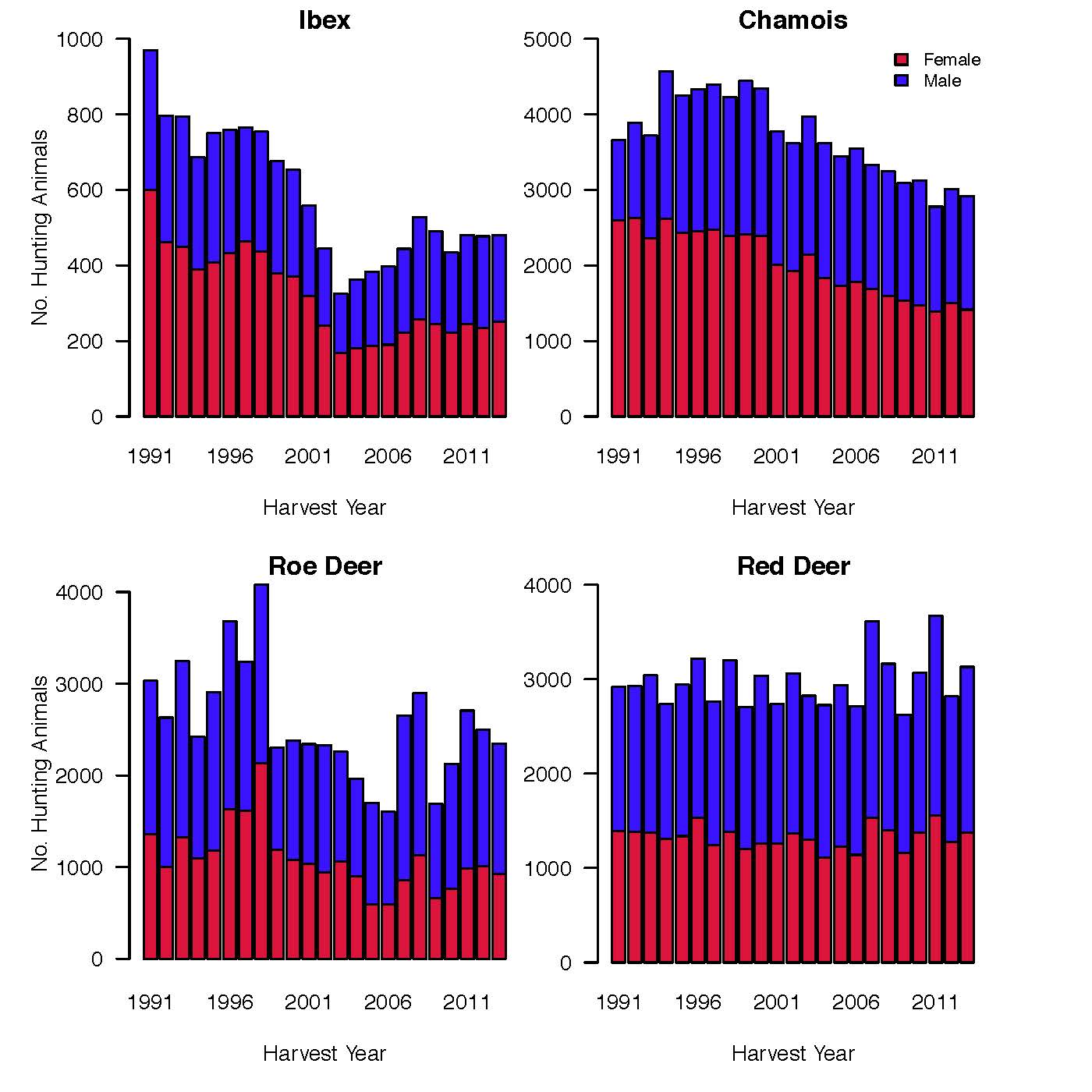


**Fig. S1.** The annual harvest of GR’s four main ungulate species between 1991 and 2013, separated into male and female proportion.


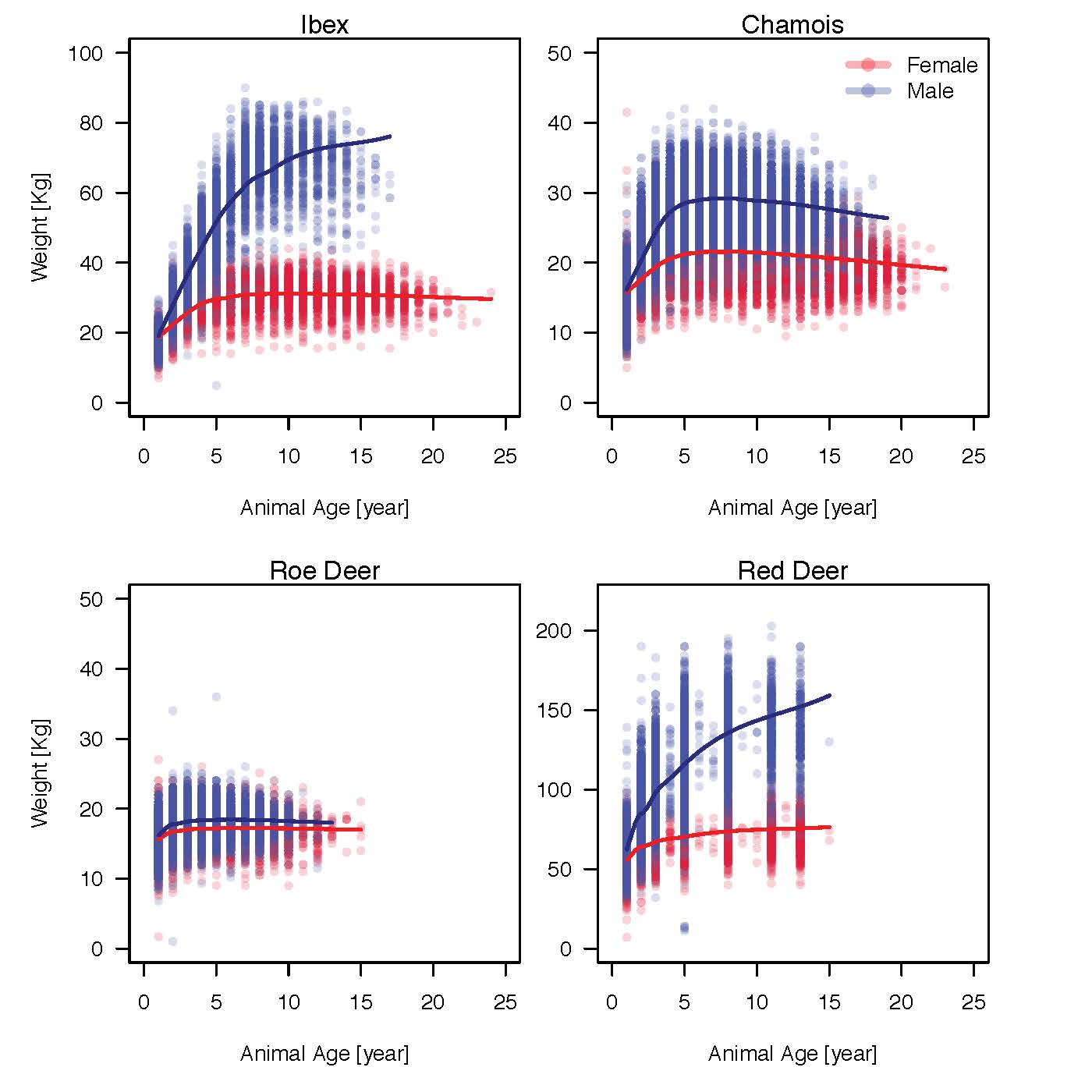


**Fig. S2.** Species-specific relationship between animal age and eviscerated body weight (EBW) of all harvested ungulates, separated into male and female proportion, and emphasized with smoothed curve fittings.


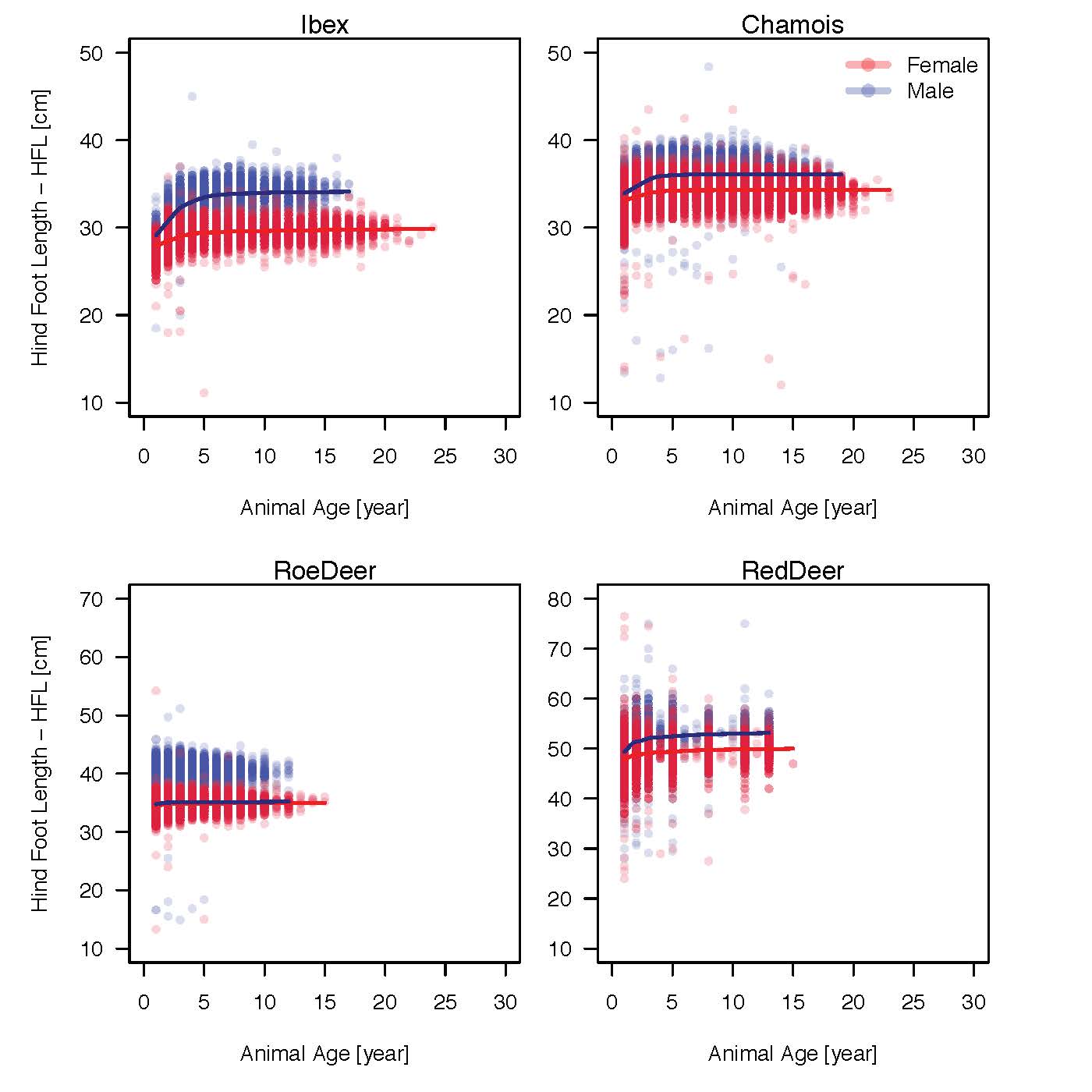


**Fig. S3.** Species-specific relationship between animal age and hind foot length (HFL) of all harvested ungulates, separated into male and female proportion, and emphasized with smoothed curve fittings.


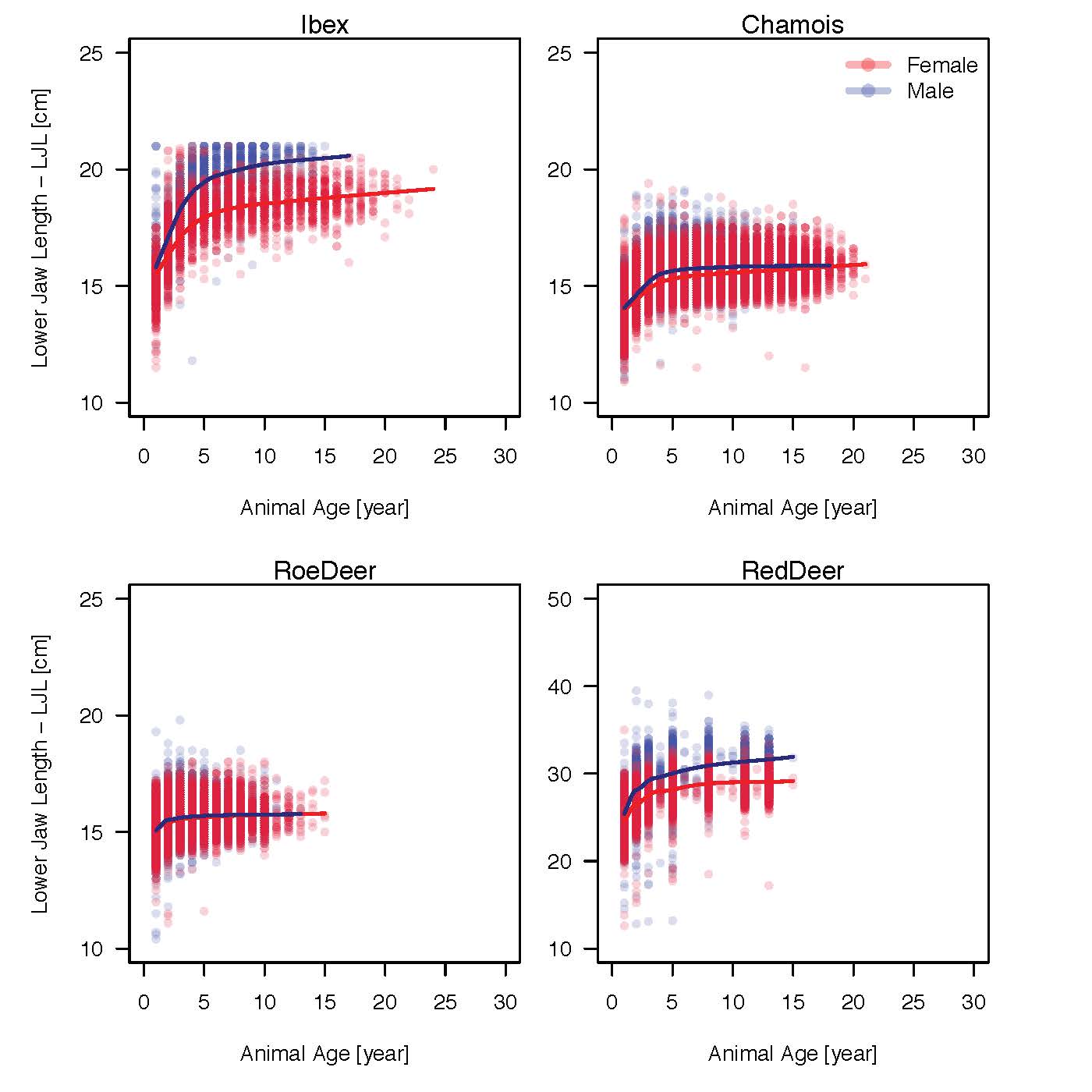


**Fig. S4.** Species-specific relationship between animal age and lower jaw length (LJL) of all harvested ungulates, separated into male and female proportion, and emphasized with smoothed curve fittings.


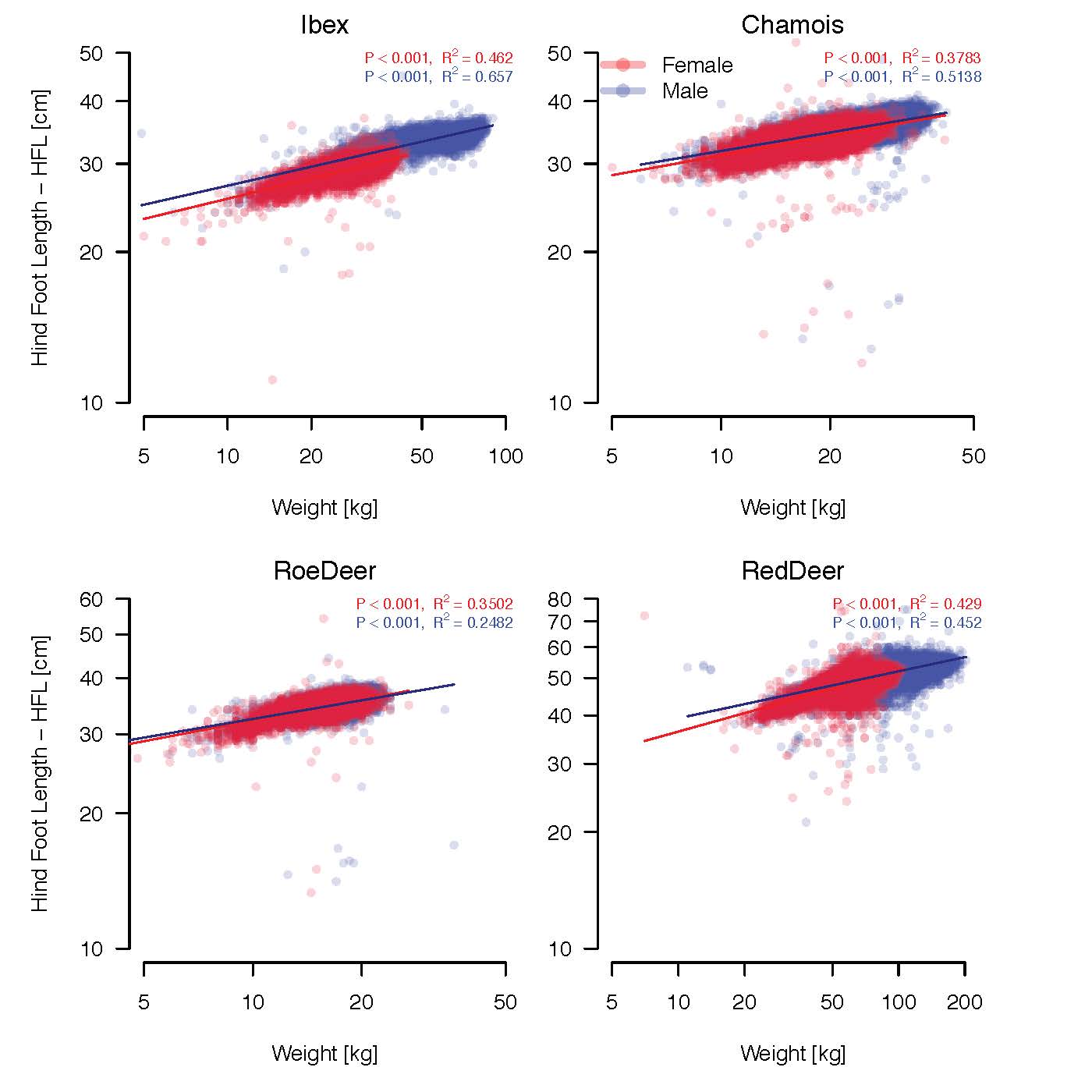


**Fig. S5.** Logged relationship between the eviscerated body weight (EBW) and hind foot length (HFL) of each ungulate separated into male and female proportions, and emphasized with smoothed curve fittings.


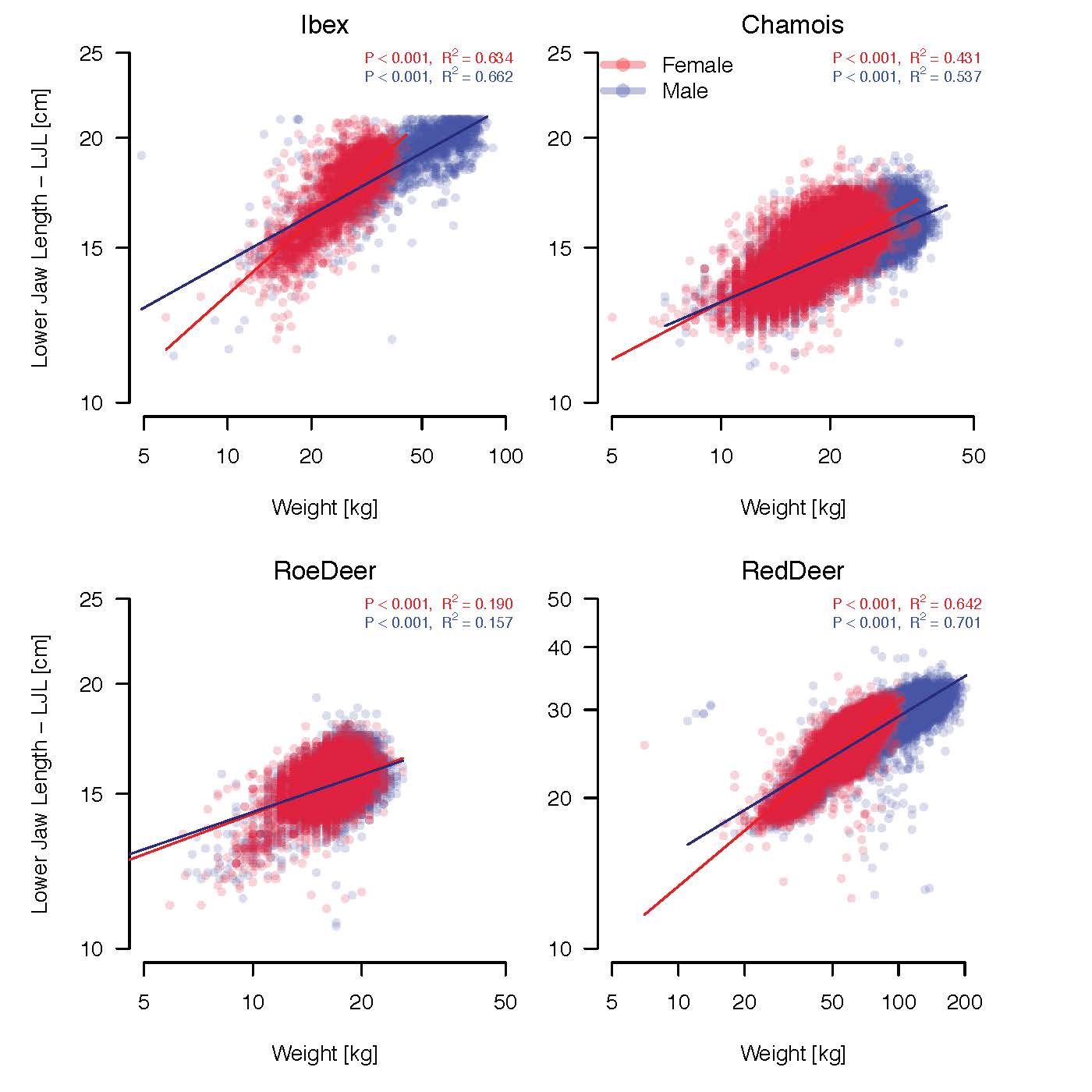


**Fig. S6.** Logged relationship between the eviscerated body weight (EBW) and lower jaw length (LJL) of each ungulate separated into male and female proportions, and emphasized with smoothed curve fittings.


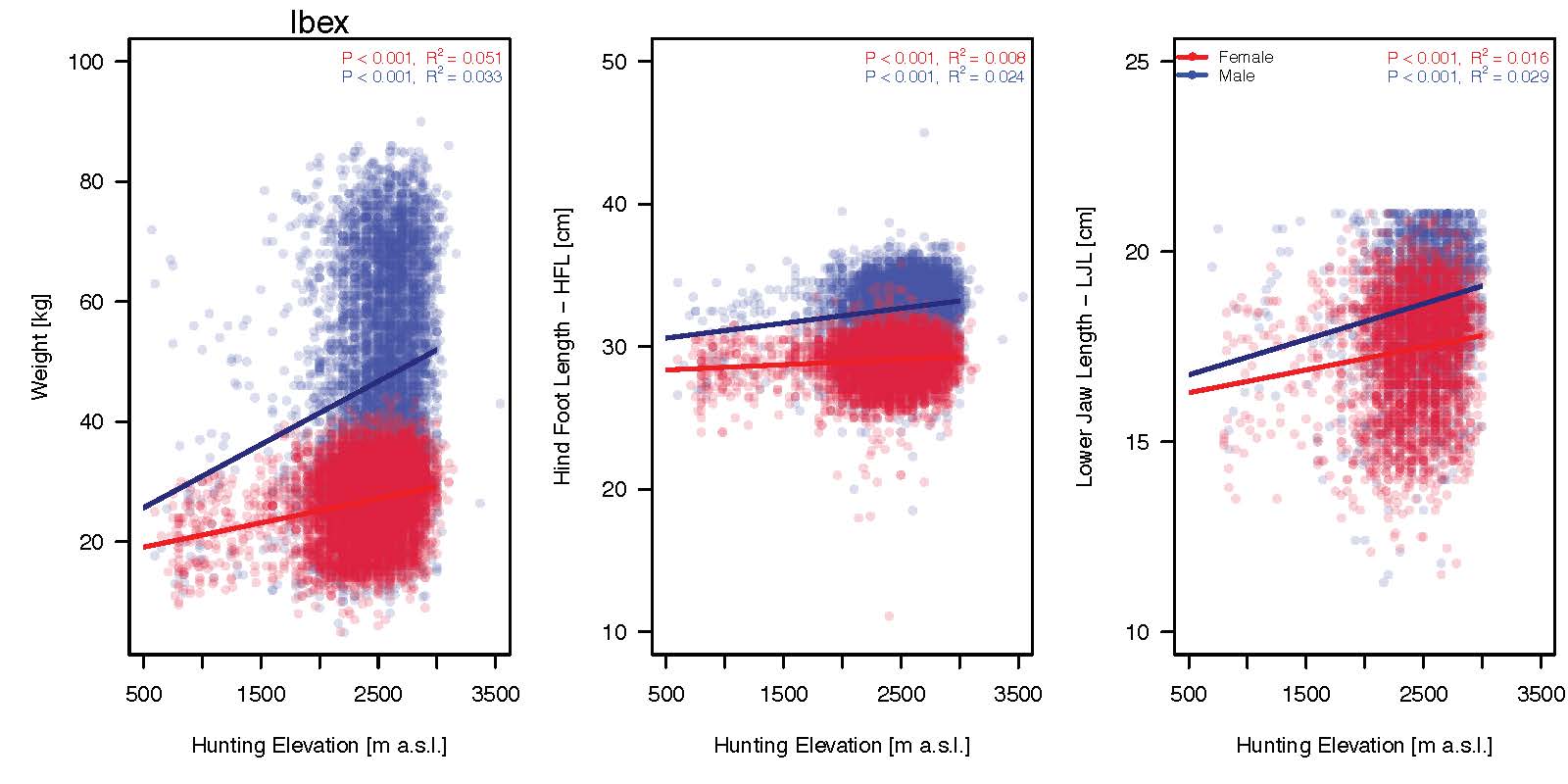


**Fig. S7.** The harvest elevation of each ibex plotted against its eviscerated body weight (EBW), hind food length (HFL) and lower jaw length (LJL), separated into male and female proportions, and emphasized with linear regression lines.


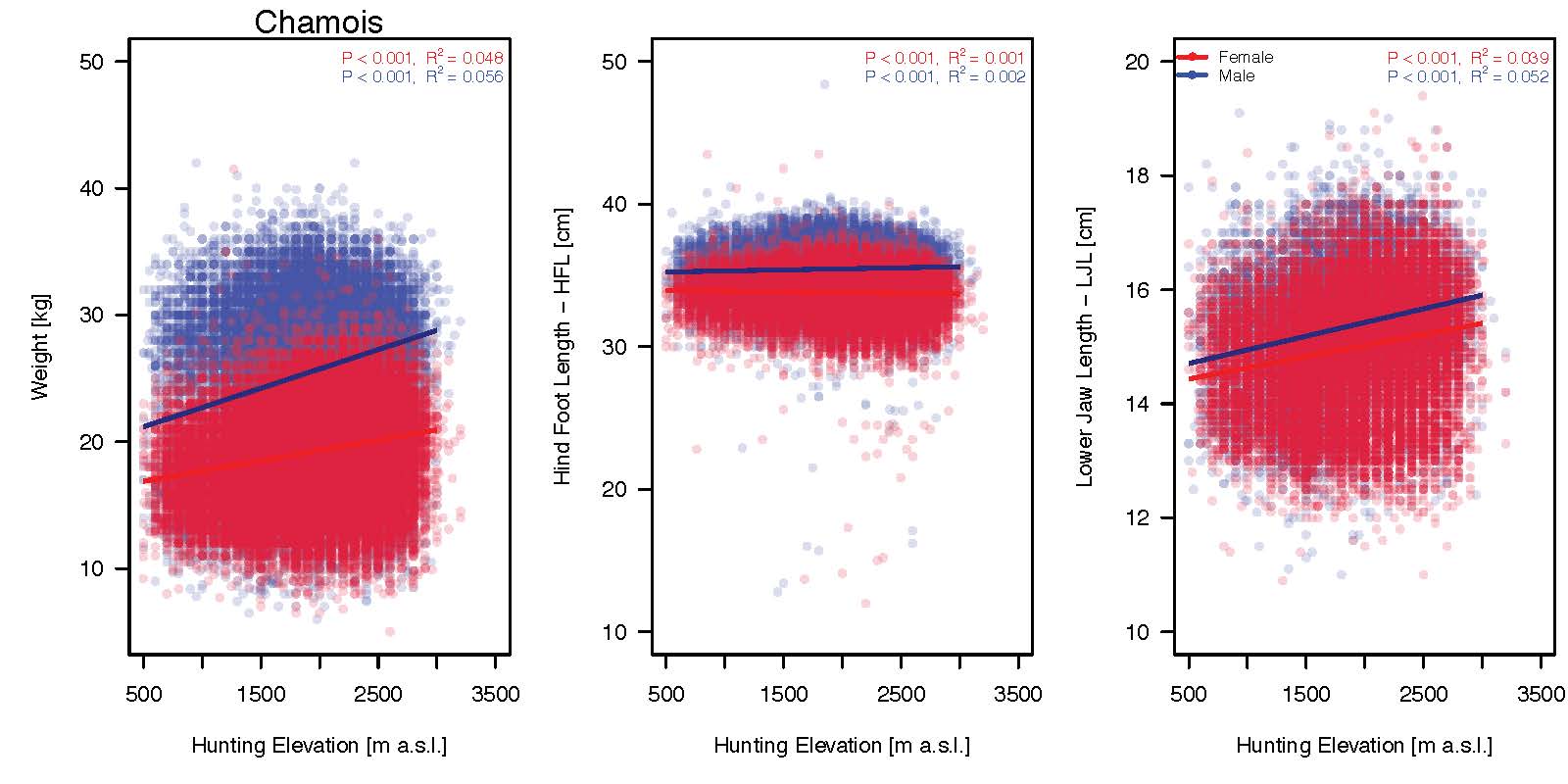


**Fig. S8.** The harvest elevation of each chamois plotted against its eviscerated body weight (EBW), hind food length (HFL) and lower jaw length (LJL), separated into male and female proportions, and emphasized with linear regression lines.


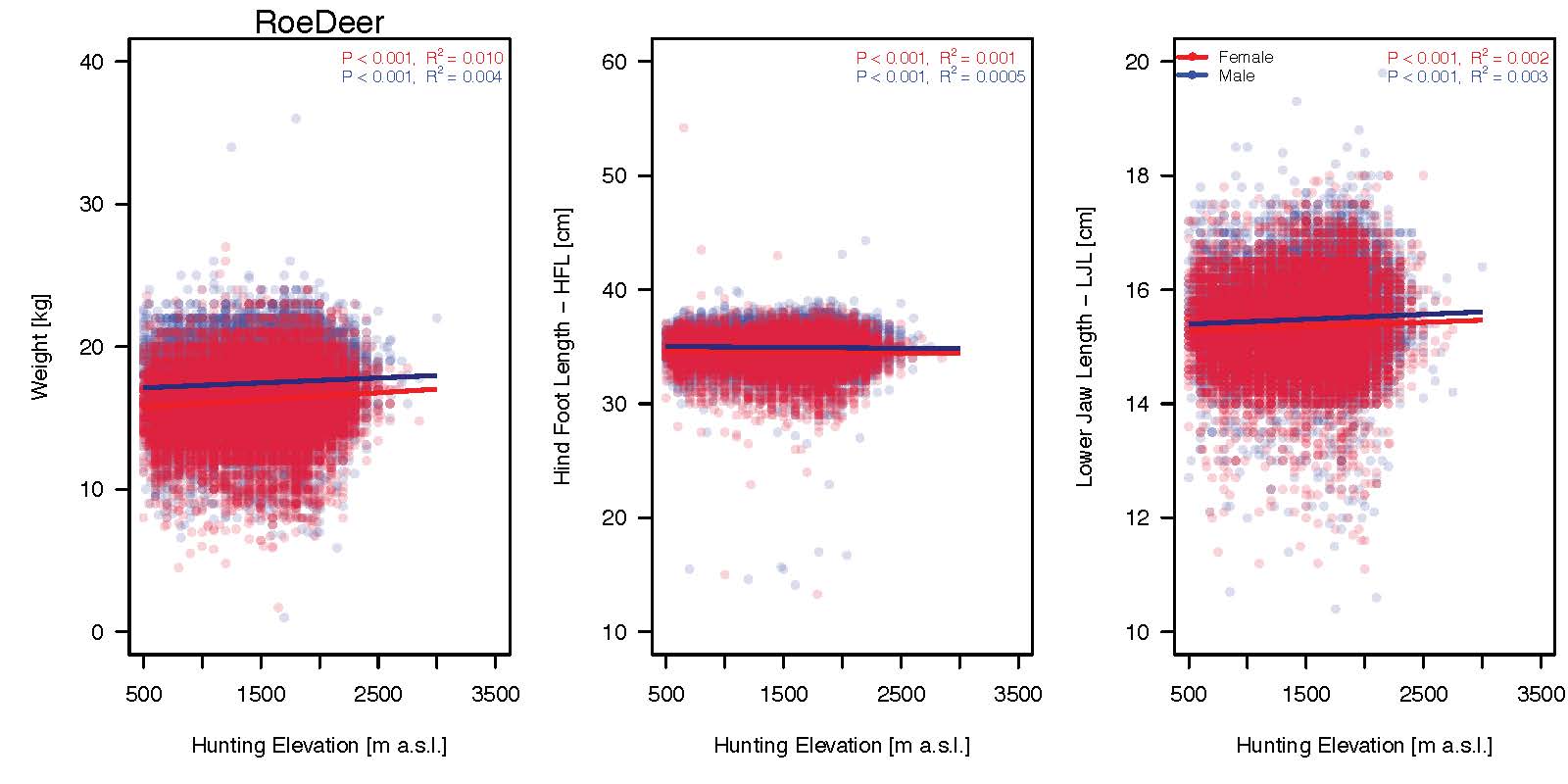


**Fig. S9.** The harvest elevation of each roe deer plotted against its eviscerated body weight (EBW), hind food length (HFL) and lower jaw length (LJL), separated into male and female proportions, and emphasized with linear regression lines.


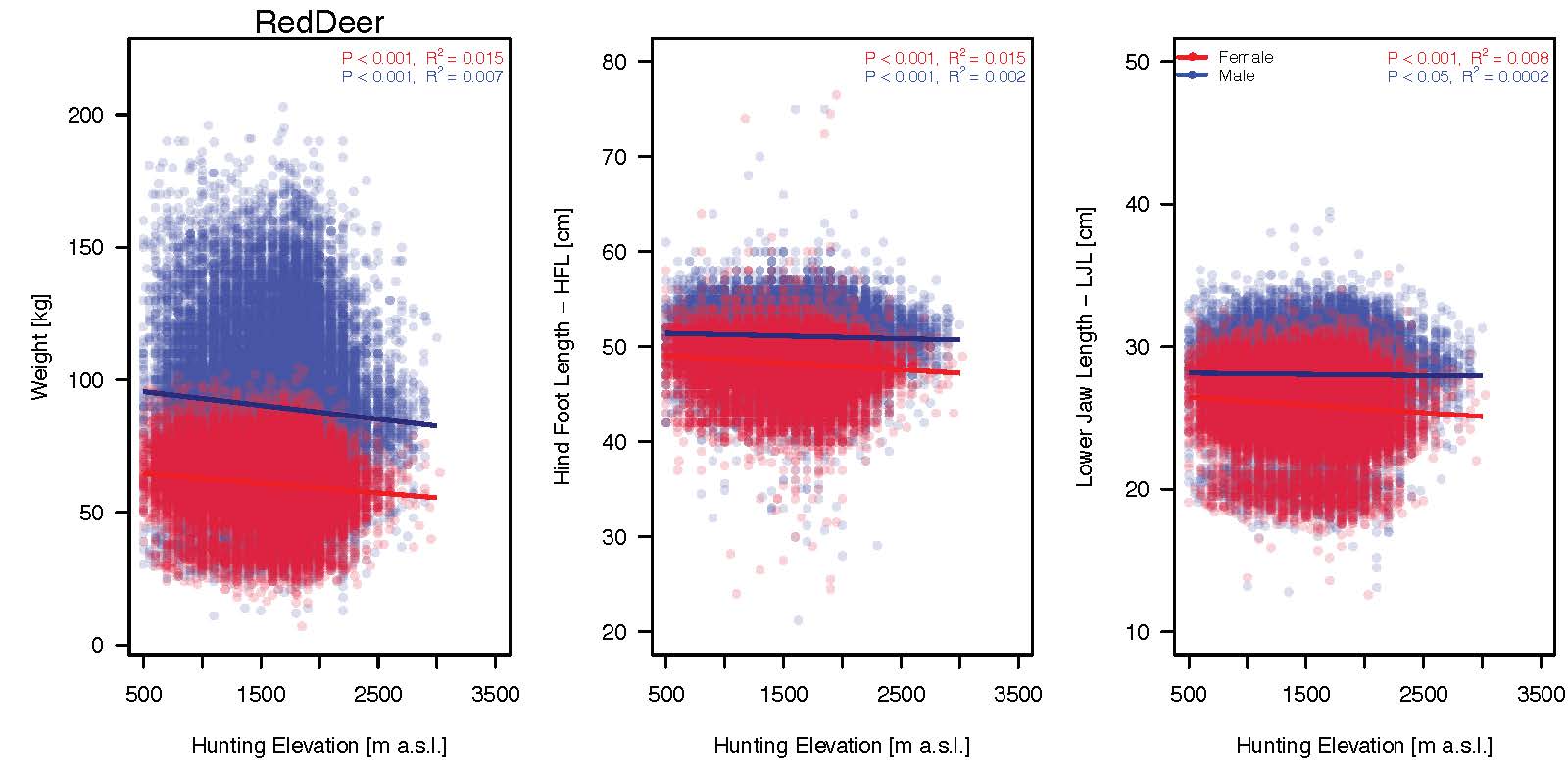


**Fig. S10.** The harvest elevation of each red deer plotted against its eviscerated body weight (EBW), hind food length (HFL) and lower jaw length (LJL), separated into male and female proportions, and emphasized with linear regression lines.
